# Stabilization in chaotic maps using hybrid chaos control procedure

**DOI:** 10.1016/j.heliyon.2024.e23984

**Published:** 2024-01-08

**Authors:** Ashish, Mohammad Sajid

**Affiliations:** a Department of Mathematics, Government College Satnali, Mahendergarh, 123024, India; b Department of Mechanical Engineering, College of Engineering, Qassim University, Buraydah, 51452, Saudi Arabia

**Keywords:** Nonlinear systems, Numerical results, Chaos control, Bifurcation plot, Lyapunov exponent

## Abstract

Much attention have been devoted to control of chaos in nonlinear system in the last few decades and several control procedures have been derived to find the stability target in difference and differential equations. In this study, a novel hybrid chaos control procedure is derived which allows to stabilize the chaos in most accepted discrete chaotic equations of population growth models about the globally accepted stable equilibrium. Since the system depends on the parameters *κ*, *α*, and *r*, the chaos in the given system may be stabilized in different fixed points states of order *p*, when it is kicked with the parameter *κ*. From this point of view, the procedure is simple, flexible, and gives the advantage to take the numerous parameter values to reach the demanded stability in periodic states of order *p*. This hybrid approach to control makes it novel as compared to existing methods. Further, we provide the geometrical interpretation followed by a few examples, control curves, bifurcation plots, time-series plots, and Lyapunov exponent to illustrate our numerical results.

## 1 Introduction

Control of chaos, in short, is a term which exhibit stability in aperiodic states of a system and has been the central issue of research in control and chaos theory modeled by nonlinear difference and differential equations. It is comprehensively used in almost every science such as mathematics, physics, medical science, engineering, etc and the stability in chaos can be useful where it gives destructive effect. Such a property has been discussed at several platforms of control theory. Ott et al. [1] first introduced the concept of control using feedback procedures which finds the stabilization in aperiodic orbits embedded in the irregular regime of the dynamics of nonlinear system. But the modern study on control completely depends on the numerical and experimental work of Pyragas [2]. Matias and Guemez [3] introduced a special control method which stabilizes chaos using proportional feedback pulse control technique in system variable. Afterward, several control methods were introduced using different techniques such as hybrid control method [4], periodic proportional pulses [5], stabilization using predictive control procedure [6], stabilization through constant feedback system [7], delayed feedback control method [8], entropy control techniques [9], [10].

In the last few decades, discrete control of chaos has played a vital role in nonlinear systems and is considered on the top of the research in dynamical systems. Therefore, they are adopted by various scientific disciplines like cryptography, traffic control models, neural network systems, secure communication, nonlinear optical systems, electric circuits, etc. In 1991, Hunt [11] studied on the modification to the results of Ott et al. [1] and determined the stability in the chaotic orbits generated by diode resonators in electric circuits. Again, in 1991, Azevedo et al. [12] used MPSW instability method to control chaos using small perturbations in the given parameter and Ditto et al. [13] also found that chaos can be controlled using the method of Ott et al. which requires small time perturbations and does not claim any model equations. Later on, Gerfinkel et al. [14] determined the stabilization in irregular orbits using the sensitivity conditions and then the same procedure was used to control cardiac arrhythmias produced in Rabbit ventricle. Further, the control to chaos method was used to find the control in aperiodic orbits of order one, two and four in prototype models of chemical chaos by Peng et al. [15]. In 1994, Sckiff et al. [16] examined that the chaos control techniques can be used in vitro to increase the stability in periodicity bursting neuronal behavior and the system can be converted into low periodicity. Similarly, Sinha [17] demonstrated that the controlling techniques can be implemented in various applications of biological systems. The traffic flow models also admit nonlinear dynamical behavior, therefore, the irregularity in the traffic flow models can also be controlled by various control techniques. The dynamical behavior in the traffic transportation problems first studied by Jarett and Zhang [18] in trip distribution. The analysis in this model gives the evidence of the chaos and periodicity in traffic flow. In 1990, Disbro and Frame [19] examined the chaotic behavior and also introduced a methodology to detect the chaos in traffic flow models. Furthermore, for a huge knowledge on control theory, authors may refer to Morgul [20], Mirus and Spott [21], Boccaletti et al. [22], Shang et al. [23], Holmgren [24], Devaney [25], Renu et al. [26].

Since 2016, Braverman et al. [27] in a series of research articles examined the stability in aperiodic states of difference equations using noisy PF control techniques, noisy prediction based technique [28], stochastic stabilization method [29], noisy proportional feedback technique [30]. In 2021, Hansen et al. [31] proposed a novel scheme to stabilize the aperiodic orbits of a dynamical system using suitable modulated control parameter. A temporary adaptive control technique based on short impulses was developed to stabilize the divergence in generalized logistic map by Lu et al. [32] in 2018. Baleanou et al. [33] found the stability in Caputo delta fractional difference map using direct theorem on discrete fractional Lyapunov method. In 2017, Z. Wei et al. [34] examined the multistability and coexistence in autonomous dynamo system in three dimensions such as limit cycles, equilibrium and hidden chaotic attractors. Again, in 2019 various examples of chaos was investigated for chameleon-like chaotic system by Z. Wei et al. [35]. An inquiry about the Jacobi stability of 5D self-exciting homopolar disc dynamo system based on differential method was carried out in 2022 [36]. Further, a classical Melnikov method for heteroclinic orbits was studied theoretically for a class of hybrid piece-wise smooth systems by Z. Wei et al. [37]. Moreover, they investigated the complicated dynamics in piecewise-smooth system with and without noise excitation under extended maps and non-autonomous periodic and damping perturbations by new method. In 2019, the control of chaos using superior feedback method was studied by Ashish et al. [38] and also in a series of article they examined the dynamic properties in various generalized logistic maps (see also Ashish et al. [39], [40], [41], [42]). In a series of articles, M. Sajid et al. [43], [44], [45] studied the chaotic properties of two-parameter functions with the logarithmic system, control of chaos in fractional order maps, and control in microscopic chaos using adaptive control technique. In 2023, F. Wang et al. [46] examined the coexistence of heteroclinic cycles in 3D piecewise dynamical systems with three discontinuous switching manifolds using various mathematical analysis. They also presented the occurrence of two horseshoes and studied the conditions for chaotic invariant sets.

This article is a one-step forward: by using the hybrid chaos control procedure in superior dynamical system (1) depending on the kicked parameter *κ*, logistic parameter *r* and Mann parameter *α* the stabilization in chaotic maps is established. This research is concluded in 4 sections. Section 1 presents a short review of the literature on controlling chaos in scientific and technical applications. In Section 2, we obtain a hybrid chaos control procedure with the composition of a superior feedback system and unidimensional chaotic maps. Chaos control is performed in Section 3 by using a hybrid control procedure followed by functional control curves, time-series curves, bifurcation plots, and maximum Lyapunov exponent. All the results are summarized in Section 4.

## 2 Hybrid chaos control procedure

A hybrid chaos control procedure is derived for discrete one-dimensional maps, which leads to strong stability in chaotic regimes through periodic orbits of order *p*. Further, it is mentioned that the procedure is a composite of nonlinear one-dimensional maps and the superior feedback system charged by the kicked control parameter *κ*. Therefore, let us consider the following superior feedback dynamical system:

(1) $$x_{n+1}=(1-\alpha )x_{n}+\alpha \phi_{r}(x_{n})=M_{\alpha ,r}(x_{n}),\;n\in N,$$

where the state vector $x_{n}\in [0,1]$, the control parameter α∈[0,1] and the function $\phi_{r}$ stands for the nonlinear maps defined on a closed interval *I* having fixed point $x^{⁎}$ which intersect the diagonal axis y=x, that is, $\phi_{r}(x^{⁎})=x^{⁎}$. At α=1, the given system (1) reduces into trivial one-dimensional system $x_{n+1}=\phi_{r}(x_{n})$. Now, we recall the definition given in [47] for stability. That means, if $x^{⁎}$ is a periodic of order p≥1 for a given superior dynamical system (1), that is, $M_{\alpha ,r}(x)$, then, $x^{⁎}$ is spoken locally stable if it fulfill:

(2) $$|\frac{d}{dx}M_{\alpha ,r}^{p}(x^{⁎})|<1.$$

Here, it is proposed to control of chaos in superior dynamical system (1) for discrete one-dimensional maps. Therefore, the superior iterative orbit $\left\{x_{n}\right\}$, where n∈N is charged by the kicked parameter κ∈(0,1) once at every *p* iterations of the system (1) as given by:

(3) $$x_{n+1}=\kappa M_{\alpha ,r}^{p}(x_{n})=G(x),\text{that is},\;x_{n+1}=\kappa [M_{\alpha ,r}(M_{\alpha ,r}(M_{\alpha ,r}(M_{\alpha ,r}(x)...p-times)))]=G(x),$$

where *κ* is taken as kicked parameter, *n* represents the multiple of *p* and $M^{p}$ stands for the $p^{th}$ iteration of the system *M* with itself *p* times. Then, the fixed point solutions of periodic order 1,2,3,…,p-times of the equation (3) are found as:

(4) $$\kappa M_{\alpha ,r}^{1}(x^{⁎})=x^{⁎}\;\text{(for period-1),}\;\kappa M_{\alpha ,r}^{2}(x^{⁎})=x^{⁎}\;\text{(for period-2),}\;\kappa M_{\alpha ,r}^{3}(x^{⁎})=x^{⁎}\;\text{(for period-3),}\vdots \;\vdots \text{and,}\;\kappa M_{\alpha ,r}^{p}(x^{⁎})=x^{⁎}\;\text{(for period-}p\text{).}$$

Thus, the stability of $x^{⁎}$ is determined by:

(5) $$|\kappa \frac{d}{dx}M_{\alpha ,r}^{p}(x^{⁎})|<1,\text{that is,}\;-1<\kappa \frac{d}{dx}M_{\alpha ,r}^{p}(x^{⁎})<1,$$

where the derivative is taken for the fixed point of $p^{th}$ order. Further, it is noticed that the stationary point for G(x) is the stable periodic stationary point of order *p* for the superior dynamical system (1) which is charged by the kicked parameter *κ*.

Now, if we take an original superior dynamical system (1) which is chaotic in its full range of growth rate parameter *r*, then, the question arises, “How to stabilize the chaos by kicking its orbits in to the stable periodic orbits of order *p*”. Therefore, first we find the parameter *κ* and then the fixed point of order *p* using equations (4) and (5). Let us define the function as follows:

(6) $$F_{\alpha ,r}^{p}(x)=\frac{x}{M_{\alpha ,r}^{p}(x)}\frac{d}{dx}M_{\alpha ,r}^{p}(x)$$

and is named as the hybrid control unit for the stability in chaos. Then, the stability is measured using the control unit (6) through the following stability condition:

(7) $$-1<F_{\alpha ,r}^{p}(x)<1,$$

and the fixed points satisfying the condition (7) will stabilize the complete chaos in the original one-dimensional dynamical system (1). Further, for the fixed points $x^{⁎}$ which satisfies the condition (7), the kicked parameter *κ* is determined as follows:

(8) $$\kappa =\frac{x^{⁎}}{M_{\alpha ,r}^{p}(x^{⁎})}.$$

Thus, for each variable *x* which satisfies (7) there corresponds an individual kicked parameter *κ* which ensures the stability in an original nonlinear dynamical system (1). The condition (7) is the major backbone of this research work, if *x* satisfies the condition (7), then there corresponds a kicked parameter *κ* in (8) which stabilize the dynamics in the superior dynamical system (1) at a period-*p* through the hybrid control system and passing through the given point *x*.

In further sections, we will take the chaotic map rx(1−x) as a typical example to demonstrate the control of chaos using hybrid chaos control procedure. The stability is described followed by bifurcation plots, time-series plots, and the maximum Lyapunov exponent spectrum.

## 3 Controlling chaos via hybrid chaos control procedure

As studied in the above section, the chaos in nonlinear one-dimensional equations may also be stabilized using hybrid chaos control procedure. In this procedure an original one-dimensional dynamical system is charged with the kicked parameter *κ* in which the unstable periodic orbits of order *p* are stabilized in stable periodic states of an original system, that is, no chaos occurs in the system. Then, we take

(9) $$\phi_{r}(x)=rx(1-x),$$

where r∈[0,4] and x∈[0,1]. Then, substituting (9) in the given superior dynamical system (1), we get the novel modified form as follows:

(10) $$M_{\alpha ,r}(x)=x-\alpha x(1-r)+\alpha rx^{2},$$

where α∈(0,1), x∈[0,1] and the logistic parameter *r* lies between 0 and $r_{max}$. Here, the parameter $r_{max}$ stands for the maximum growth-rate value which depends on the parameter *α*. In particular, when α=0.9 the logistic parameter *r* lies between 0 and 4.22. Further, the equation (10) has the fixed point $x^{⁎}=1-\frac{1}{r}$ of period one and the fixed points

(11) $$x_{1}^{⁎}=\frac{2r\alpha -r\alpha^{2}+r^{2}\alpha^{2}-r\alpha \sqrt{-4+\alpha^{2}-2r\alpha^{2}+r^{2}\alpha^{2}}}{2r^{2}\alpha^{2}},$$

(12) $$\text{and}\;x_{2}^{⁎}=\frac{2r\alpha -r\alpha^{2}+r^{2}\alpha^{2}+r\alpha \sqrt{-4+\alpha^{2}-2r\alpha^{2}+r^{2}\alpha^{2}}}{2r^{2}\alpha^{2}}$$

of period two. In case of α=0.9, it is examined that the fixed state $x^{⁎}$ of period one is always unstable when r≥3.2199 and the periodic fixed states $x_{1}^{⁎}$ and $x_{2}^{⁎}$ of period-2 are always unstable when r>3.7269 and finally approaches to chaos. Similarly, the higher order periodic fixed points also get unstable when *r* approaches beyond 3.8554.

**Figure 1:**
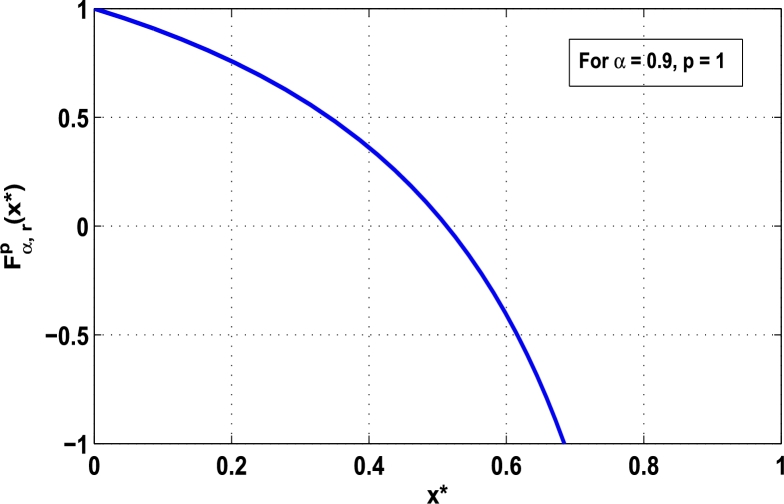
Control curve at *p* = 1 for the system *M*_*α*,*r*_(*x*) when *r* = 4.22.

**Figure 2:**
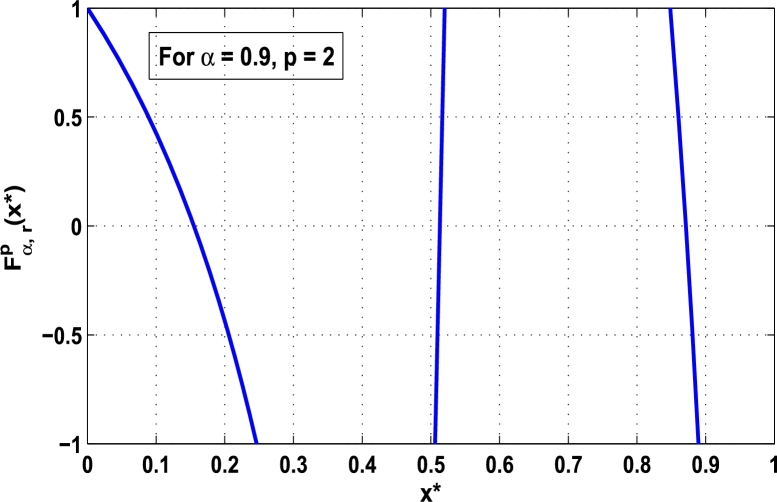
Control curve at *p* = 2 for the system *M*_*α*,*r*_(*x*) when *r* = 4.22.

**Figure 3:**
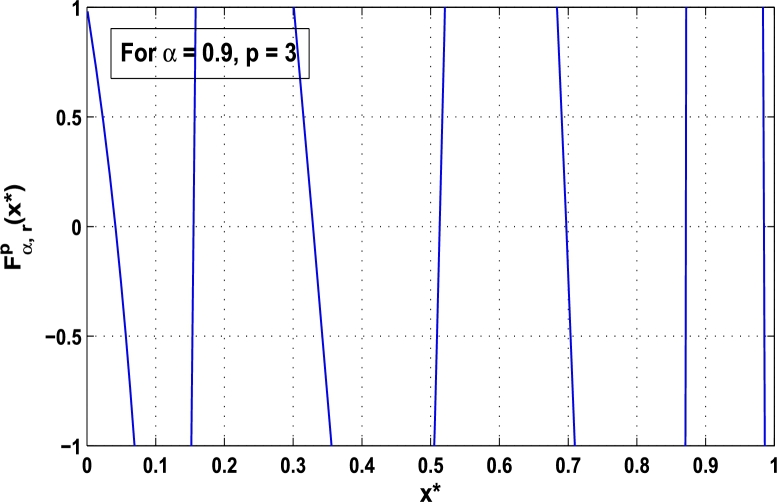
Control curve at *p* = 3 for the system *M*_*α*,*r*_(*x*) when *r* = 4.22.

**Figure 4:**
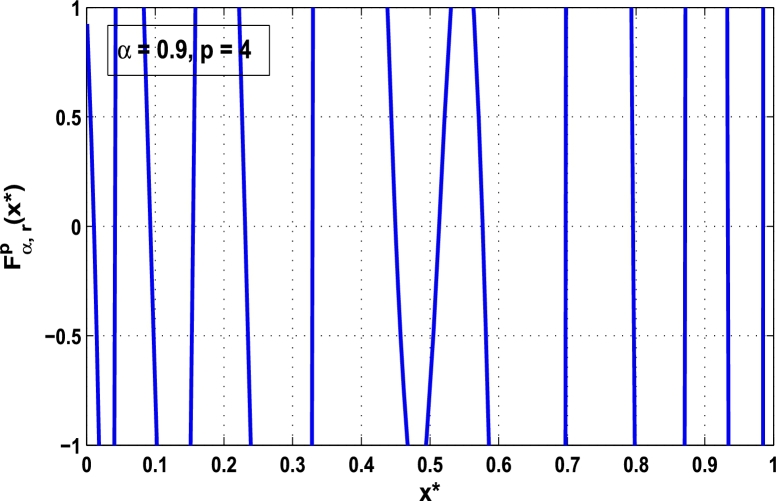
Control curve at *p* = 4 for the system *M*_*α*,*r*_(*x*) when *r* = 4.22.

**Figure 5:**
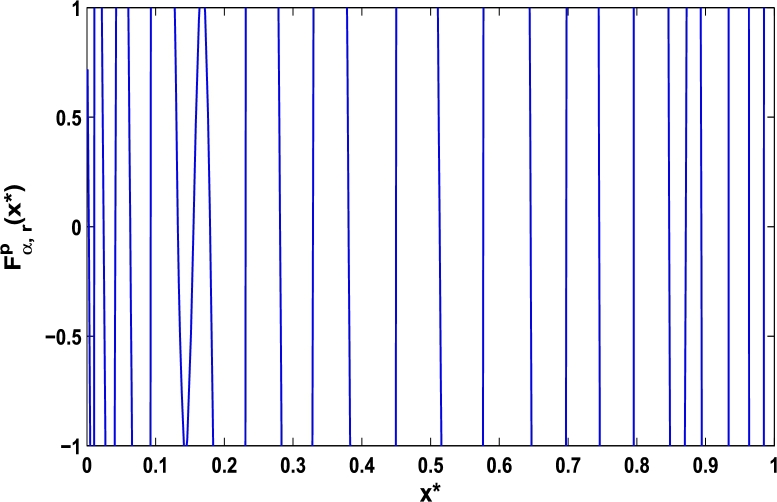
Control curve at *p* = 5 for the system *M*_*α*,*r*_(*x*) when *r* = 4.22.

**Figure 6:**
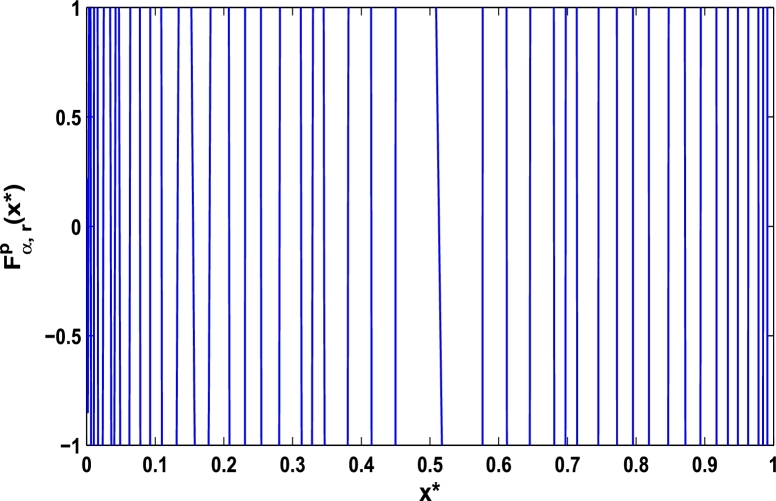
Control curve at *p* = 6 for the system *M*_*α*,*r*_(*x*) when *r* = 4.22.

Now, we determine the stability in chaos for an original superior dynamical system (10) which depends on the parameter *α*. Therefore, let us start to find the fixed points of order *p* in the closed interval [0,1] using the hybrid chaos control procedure for which the system (10) gets stability of periodic orders 1,2,… and so on. Then, we draw time-series functional diagrams for $F_{\alpha ,r}^{p}(x)\in (-1,1)$ starting from an arbitrary point x∈[0,1]. Figure 1, Figure 2, Figure 3, Figure 4, Figure 5, Figure 6 show the functional plots for $F_{\alpha ,r}^{p}(x)$ versus x∈[0,1] for the periodicity *p* of order 1, 2, 3, 4, 5, and 6 and is bounded to the area of the system between −1 and 1. Fig. 1 shows that at α=0.9 and p=1, the iterative orbit of the system can be stabilized at every point *x* lies between 0 and 0.68 which are the states of period-1. When α=0.9 and p=2, the iterative orbits may be established in three areas of *x* values in which the two ranges are determined as 0<x≤0.24 and 0.81≤x≤0.88 as shown in Fig. 2. When p=3 the seven ranges of *x* values are examined as shown in Fig. 3. The few ranges in which the system is stabilized are 0<x≤0.007, 0.26≤x≤0.35, 0.66≤x≤0.70, and 0.982≤x≤0.986. The range of *x* for p=4 is determined as 0.5≤x≤0.57 as shown in Fig. 4. Similarly, Figure 5, Figure 6 also show that for p=5 and 6 the iterative orbits are also stabilized for various ranges of *x* between 0 and 1.

**Figure 7:**
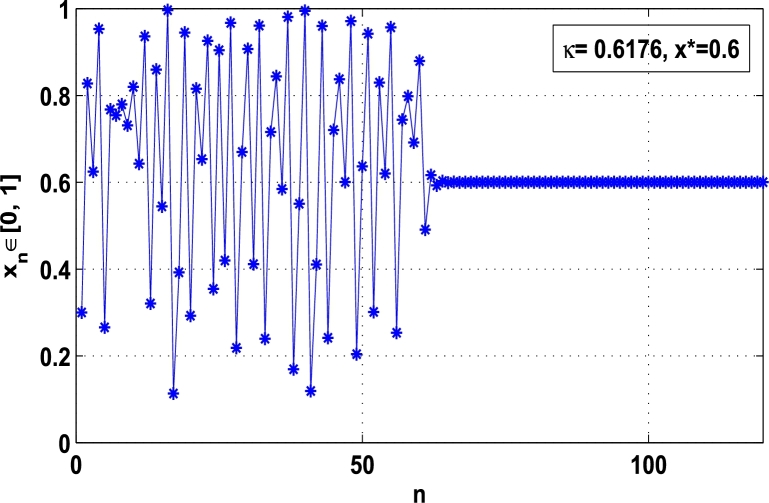
Controlling superior system *M*_*α*,*r*_(*x*) in fixed point *x*^⁎^ = 0.6 of periodic order-1.

**Figure 8:**
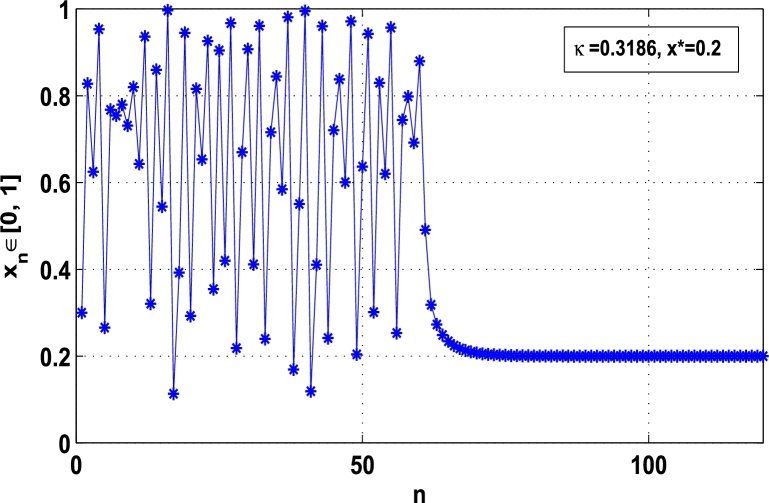
Controlling superior system *M*_*α*,*r*_(*x*) in fixed point *x*^⁎^ = 0.2 of periodic order-1.

**Figure 9:**
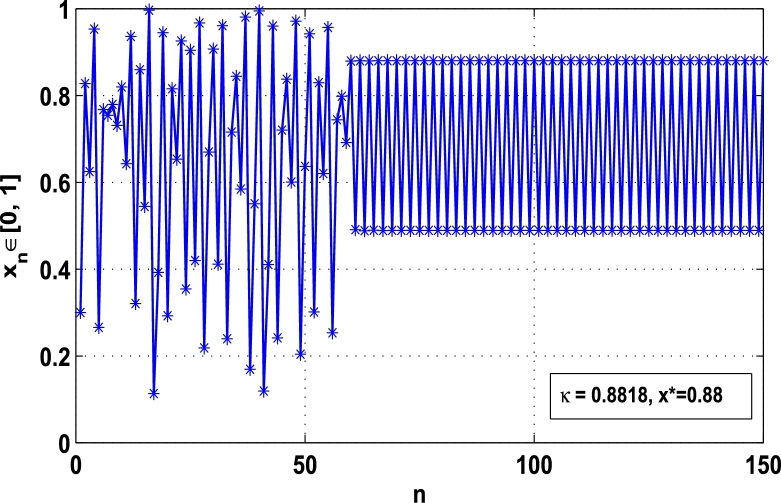
Controlling superior system *M*_*α*,*r*_(*x*) in fixed point *x*^⁎^ = 0.88 of period-2.

**Figure 10:**
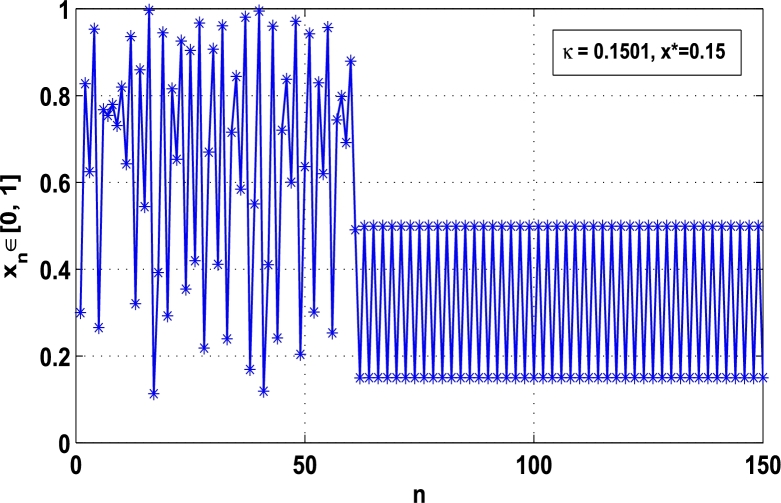
Controlling superior system *M*_*α*,*r*_(*x*) in fixed point *x*^⁎^ = 0.15 of period-2.

**Figure 11:**
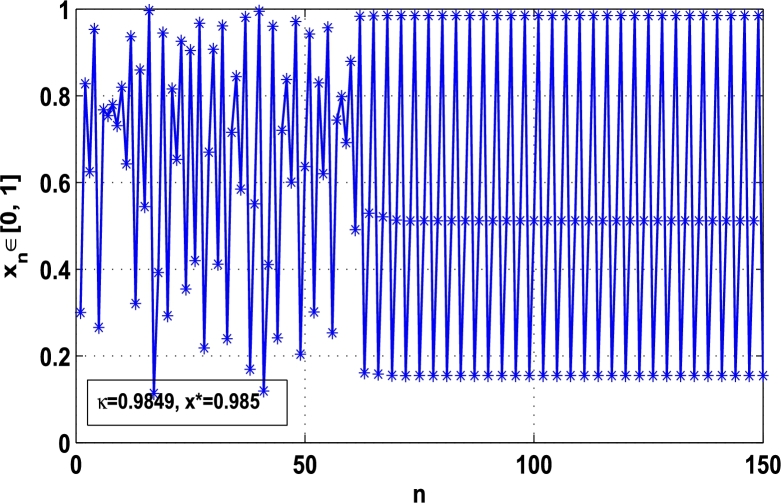
Controlling superior system *M*_*α*,*r*_(*x*) in fixed point *x*^⁎^ = 0.985 of period-3.

**Figure 12:**
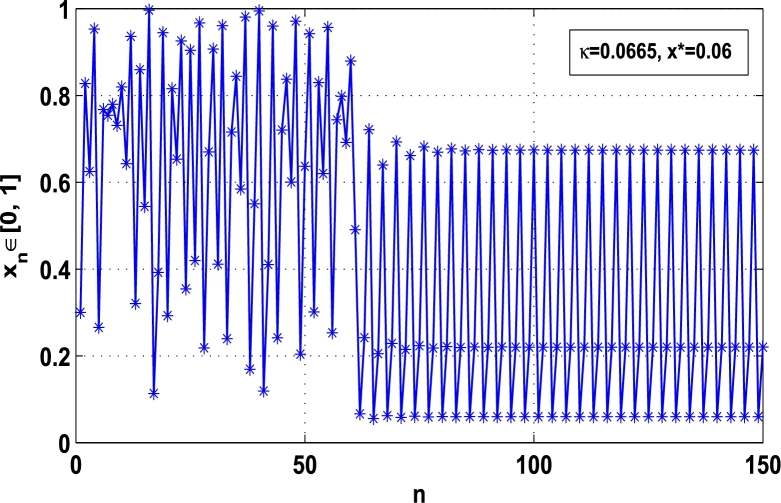
Controlling superior system *M*_*α*,*r*_(*x*) in fixed point *x*^⁎^ = 0.06 of period-3.

**Figure 13:**
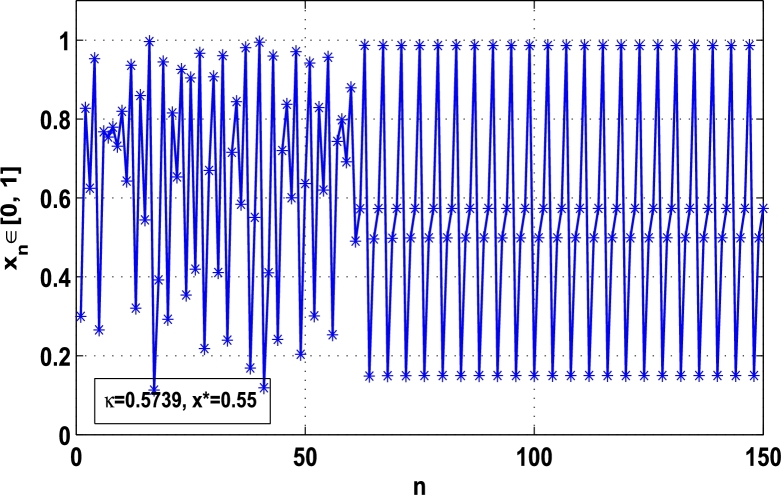
Controlling superior system *M*_*α*,*r*_(*x*) in fixed point *x*^⁎^ = 0.55 of period-4.

**Figure 14:**
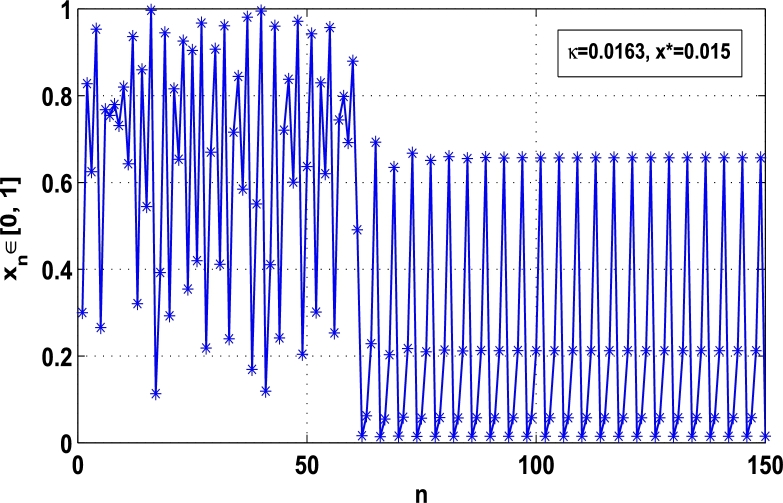
Controlling superior system *M*_*α*,*r*_(*x*) in fixed point *x*^⁎^ = 0.015 of period-4.

Moreover, it is an interesting procedure in which the iterative trajectories are stabilized at the fixed points of periodic orders 1,2,… and so on. Figure 1, Figure 2, Figure 3, Figure 4, Figure 5, Figure 6, determine the regime of arbitrary points of order *p* in the closed interval [0, 1] which satisfies the stability condition (7). Therefore, for each point from the region we check the condition (7) for the periodicity *p* of order 1 to 6. If the condition (7) is satisfied we operate equation (8) to determine the factor *κ* and then apply the control immediately. For these values of $x^{⁎}$ and *κ* the original dynamical system (10) gets stability in fixed points of periodic order 1 to 6 as shown in Figure 7, Figure 8, Figure 9, Figure 10, Figure 11, Figure 12, Figure 13, Figure 14. When the original system (10) is kicked with the parameter κ=0.6176 the unstable trajectory of the system (10) approaches to the stable trajectory of $x^{⁎}=0.6$ in r∈[0,4.22] as shown in Fig. 7 and Fig. 8 gives the stability when the orbit is kicked with the parameter κ=0.3186 once at every $1^{st}$ iteration.

Figure 9, Figure 10 show that the orbit of the system approaches to periodic fixed points $x^{⁎}=0.88$ and 0.15 of order 2 when they are kicked with the parameter κ=0.8818 and 0.1501 once at every $2^{nd}$ iterations, respectively. The trajectories approach to period 3 fixed points $x^{⁎}=985$ and 0.06 when they are controlled with the parameter κ=0.9849 and 0.0665 as shown in Figure 11, Figure 12, respectively. Finally, the system is stabilized at period 4 at $x^{⁎}=0.55$ and κ=0.5739 as shown in Fig. 13 and at $x^{⁎}=0.015$ and κ=0.0163 as shown in Fig. 14.

### 3.1 Controlling chaos and period-doubling bifurcations

In this section, we deal with the control of chaos and period-doubling bifurcations using hybrid chaos control procedure. As discussed in earlier sections, the chaos in nonlinear one-dimensional maps is stabilized using the hybrid chaos control procedure for some particular values of the kicked parameter *κ* depending on the fixed points of periodic order *p* which satisfies the condition (7). Therefore, taking some particular values of the parameter *κ* the chaotic regime is stabilized to the fixed state $x^{⁎}$ using bifurcation plot in the full range of parameter r∈[0,4.22] and α=0.9.

**Figure 15:**
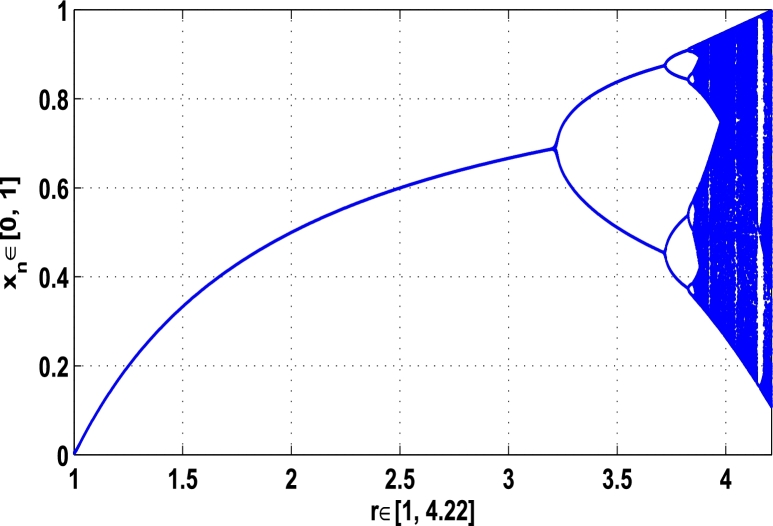
Period-doubling bifurcation plot for *M*_*α*,*r*_(*x*) when 0 ≤ *r* ≤ 4.22 and *α* = 0.9.

Generally, as shown in Fig. 15 when the growth-rate parameter *r* lies between 0 and 3.2199 the iterative orbit in the superior dynamical system (10) approaches to stable fixed points of period-1, when 3.2199<r≤3.7269, it converges to the stable fixed points of period-2, when 3.7269<r≤3.8554, it admits stable fixed points of higher orders and when 3.8554<r≤4.22 the chaos occurs in the system. Therefore, it is clear that as r≅3.2199 the fixed points of period-1 start to become unstable, as r≅3.7269 the periodic points of period-2 start to become unstable, and as r≅3.8554 the iterative orbit approaches to irregularity, that means, the chaos occurs in the superior dynamical system (10).

Following example determines the stability behavior for the logistic map using superior dynamical system (1) and the hybrid chaos control procedure (6). Here, we stabilize the traditional unstable fixed and periodic points and the chaotic orbits at stabilized periodic states $x^{⁎}$ of period p≥1 depending on the kicked parameter *κ*.

Example 3.1 Let $\phi_{r}(x)=rx(1-x)$ be a classical logistic map and $M_{\alpha ,r}(x)$ be the superior dynamical system, where α∈(0,1), r∈[0,4.22], and $x^{⁎}$ be an unstable periodic fixed point of order *p* for $M_{\alpha ,r}(x)$. Then, using the hybrid chaos control procedure (6) determine the control of chaos where the iterative orbits settle down in the stability of:

**(a)** the fixed point $x^{⁎}=0.6$ of period-1, when κ=0.6176, α=0.9 and r=4.22,
**(b)** the periodic point $x^{⁎}=0.88$ of period-2, when κ=0.8818, α=0.9 and r=4.22.

*Solution.* As discussed in subsection 3.1, for the logistic map $\phi_{r}(x)=rx(1-x)$ the chaotic regime in superior dynamical system $M_{\alpha ,r}(x)$ is stabilized at periodic fixed points of period 1,2,…, and so on using hybrid chaos control procedure. That means, the system $M_{\alpha ,r}(x)$ is kicked with the parameter *κ* after each $p^{th}$ iterations, that is, $\kappa M_{\alpha ,r}^{p}(x)=G(x)$. To determine the stability in periodic orbits of order *p*, let us take the superior dynamical system

(13) $$M_{\alpha ,r}(x)=(1-\alpha )x+\alpha \phi_{r}(x),$$

where r∈[0,4.22] and α∈(0,1). Then, the two cases are examined one by one:

**(a)** Since the fixed point $x^{⁎}=0.6$ at α=0.9 and r=4.22 is assumed unstable in an original system (13) for the map $\phi_{r}(x)=rx(1-x)$. Therefore, using the hybrid chaos control procedure the periodic state $x^{⁎}=0.6$ is stabilized in the full range of the logistic parameter *r*. Then, using the procedure (6) for the periodic state of order one, let us take

(14) $$\kappa M_{\alpha ,r}(x)=\kappa [(1-\alpha )x+\alpha rx(1-x)],$$

and then we shall show that $|\kappa M_{\alpha ,r}^{\prime }(x)|<1$. Taking the derivative of (14), we obtain

(15) $$\kappa M_{\alpha ,r}^{\prime }(x)=\kappa (1-\alpha )+\kappa \alpha r(1-2x).$$

**Figure 16:**
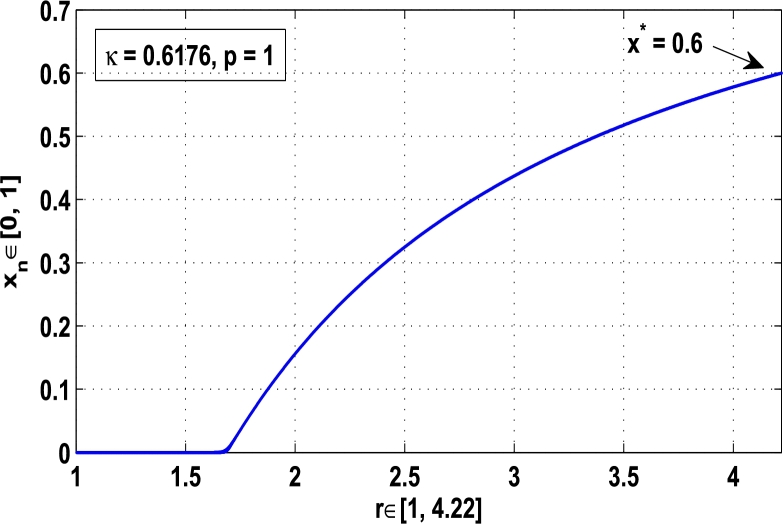
Stability at periodic fixed point *x*^⁎^ = 0.6 of period-1 when *κ* = 0.6176.

Substituting $x^{⁎}=x=0.6$, α=0.9, r=4.22 and k=0.6176 in (15), we get

(16) $$\kappa M_{\alpha ,r}^{\prime }(x)=0.6176\times (1-0.9)+0.6176\times 0.9\times 4.22(1-2\times 0.6)=0.6176\times 0.1-0.6176\times 0.9\times 4.22\times 0.2=-0.4074,$$

which implies that $|\kappa M_{\alpha ,r}^{\prime }(x)|<1$. Thus, the stationary state $x^{⁎}=0.6$ is stable for 0<r≤4.22, that is, the chaos is completely stabilized at the fixed point $x^{⁎}=0.6$ of period-1 as shown in Fig. 16.

**(b)** Here, it is proposed to examine the control of chaos at the periodic fixed point $x^{⁎}=0.88$ of period-2 at α=0.9 and r=4.22 which is unstable for an original system (13) at r=4.22. Therefore, to examine the stabilization in periodic state $x^{⁎}=0.88$ of period-2 using the hybrid chaos control procedure (6) for the fixed points of period-2, let us take

(17) $$\kappa M_{\alpha ,r}^{2}(x)=\kappa M_{\alpha ,r}(M_{\alpha ,r}(x)),$$

and then using (13), we obtain

(18) $$\kappa M_{\alpha ,r}^{2}(x)=\kappa [M_{\alpha ,r}((\alpha -1)x+\alpha rx(1-x))],\kappa M_{\alpha ,r}^{2}(x)=\kappa (x(\alpha -1)+\alpha rx(x-1))(\alpha -1)-\alpha \kappa r(x(\alpha -1)+\alpha rx(x-1))(x(\alpha -1)+\alpha rx(x-1)+1).$$

Taking the derivative of (18), we get

(19) $$\kappa M_{\alpha ,r}^{2^{\prime }}(x)=\kappa (\alpha -1)(\alpha +\alpha r(x-1)+\alpha rx-1)-\alpha \kappa r(x(\alpha -1)+\alpha rx(x-1))(\alpha +\alpha r(x-1)+\alpha rx-1)-\alpha \kappa r(x(\alpha -1)+\alpha rx(x-1)+1)(\alpha +\alpha r(x-1)+\alpha rx-1).$$

Therefore, we shall examine that $|\kappa M_{\alpha ,r}^{2^{\prime }}(x)|<1$. Substituting the periodic fixed point x=0.88 of period-2, kicked parameter κ=0.8818, the logistic parameter r=4.22, and the parameter α=0.9 in (19), we obtain

$$\kappa M_{\alpha ,r}^{2^{\prime }}(0.88)=-0.4497,\text{that is,}\;|\kappa M_{\alpha ,r}^{2^{\prime }}(0.88)|=0.4497<1.$$

**Figure 17:**
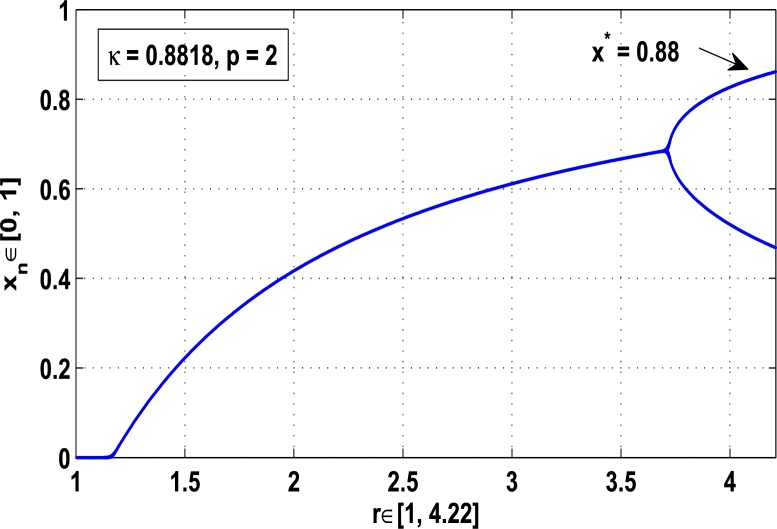
Stability at periodic state *x*^⁎^ = 0.88 of period two when *κ* = 0.8818.

**Figure 18:**
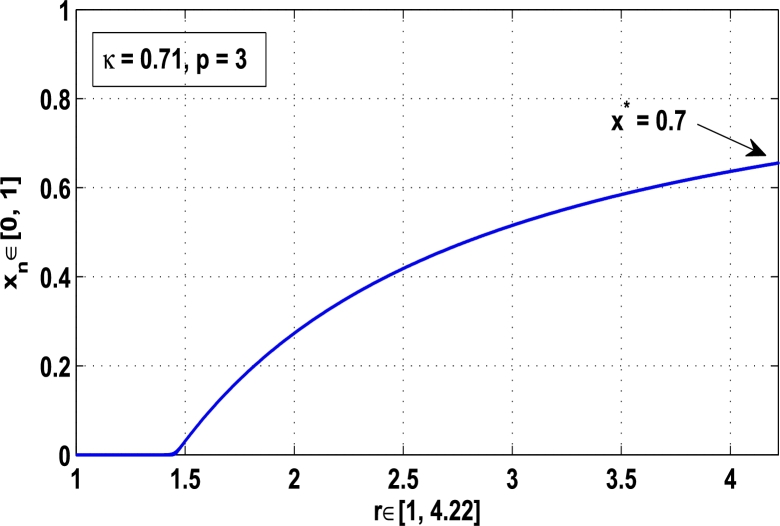
Stability at periodic fixed point *x*^⁎^ = 0.7 of period-3 when *κ* = 0.71.

Thus, the periodic fixed point $x^{⁎}=0.88$ of period-2 is stable in the full range of parameter *r*, that is, no chaos occurs in the system as shown in Fig. 17. Similarly, Fig. 18 shows that the periodic fixed point of period-3 at κ=0.71 is also stable at which the chaotic region becomes stable for r∈[0,4.22].

Figure 15, Figure 16, Figure 17, Figure 18, show the complete stability behavior in chaos using period-doubling bifurcation plots. The standard dynamics for the superior system (13) with nonlinear logistic map is shown in Fig. 15 for r∈[0,4.22]. As the hybrid chaos control procedure is imposed in the dynamical system (13), Fig. 16 gives the stability in the chaos at periodic fixed point of period-1, Fig. 17 shows the stability in the chaos at periodic state of order 2 and similarly the system is stabilized at periodic fixed point of period-3 as shown in Fig. 18.

### 3.2 Controlling chaos and maximum Lyapunov exponent

Lyapunov exponent, another important property in nonlinear dynamical systems often used whether or not the system is chaotic depending on the initial conditions. It may be determined for some points of an orbit about to the attractor to find the average Lyapunov exponent value. If the average Lyapunov value is positive then the system is said to be chaotic, if negative then the system is periodic of order-*p* and if it is zero, then the bifurcation takes place in the system. Therefore, the Lyapunov exponent procedure is adopted to determine the stability behavior in the dynamics of a nonlinear system using hybrid chaos control procedure.

For this, let us take a nonlinear map $\phi_{r}(x)$ and the hybrid chaos control procedure $G(x)=\kappa M_{\alpha ,r}^{p}(x)$ with two random initiators $x_{0}$ and $x_{0}+h$, where *h* is an arbitrary small number. The two orbits $G^{n}(x_{0})$ and $G^{n}(x_{0}+h)$ exists with respect to $x_{0}$ and $x_{0}+h$. Then, the parameter *μ* depending on the iterative orbits of $x_{0}$ and $x_{0}+h$ as n→∞ is defined as:

(20) $$\lim_{n\to \infty }e^{n\mu }=\lim_{n\to \infty }\frac{\left|G^{n}(x_{0}+h)-G^{n}(x_{0})\right|}{h}.$$

Substituting $G(x)=\kappa M_{\alpha ,r}^{p}(x)$ in (20), we obtain

(21) $$\lim_{n\to \infty }e^{n\mu }=\lim_{n\to \infty }\frac{\left|\kappa^{n}{\left(M_{\alpha ,r}^{p}\right)}^{n}(x_{0}+h)-\kappa^{n}{\left(M_{\alpha ,r}^{p}\right)}^{n}(x_{0})\right|}{h},=\kappa^{n}\lim_{n\to \infty }\frac{\left|{\left(M_{\alpha ,r}^{p}\right)}^{n}(x_{0}+h)-{\left(M_{\alpha ,r}^{p}\right)}^{n}(x_{0})\right|}{h}.$$

As n→∞ implies h→0 and the parameter *μ* is known as average Lyapunov exponent value. When the parameter μ>0, then the orbit is chaotic, and when μ≤0 the orbit is periodic of order-*p*, that is, stable system. Therefore, to examine the parameter *μ*, let us take the logarithm in (21):

(22) $$n\mu =\kappa^{n}\lim_{n\to \infty }\lim_{h\to 0}\ln\frac{\left|{\left(M_{\alpha ,r}^{p}\right)}^{n}(x_{0}+h)-{\left(M_{\alpha ,r}^{p}\right)}^{n}(x_{0})\right|}{h},\mu =\frac{\kappa^{n}}{n}\lim_{n\to \infty }\lim_{h\to 0}\ln\frac{\left|{\left(M_{\alpha ,r}^{p}\right)}^{n}(x_{0}+h)-{\left(M_{\alpha ,r}^{p}\right)}^{n}(x_{0})\right|}{h},\mu =\lim_{n\to \infty }\frac{\kappa^{n}}{n}\ln|{\left({\left(M_{\alpha ,r}^{p}\right)}^{n}\right)}^{\prime }(x_{0})|.$$

Then, using the definition of chain rule of differentiation, we can write

(23) $${\left({\left(M_{\alpha ,r}^{p}\right)}^{n}\right)}^{\prime }(x_{0})={\left(M_{\alpha ,r}^{p}{\left(M_{\alpha ,r}^{p}\right)}^{n-1}\right)}^{\prime }(x_{0}),=(M_{\alpha ,r}^{p^{\prime }}{\left(M_{\alpha ,r}^{p}\right)}^{n-1})(x_{0}).{\left({\left(M_{\alpha ,r}^{p}\right)}^{n-1}\right)}^{\prime }(x_{0}),=(M_{\alpha ,r}^{p^{\prime }}{\left(M_{\alpha ,r}^{p}\right)}^{n-1})(x_{0}).(M_{\alpha ,r}^{p^{\prime }}{\left(M_{\alpha ,r}^{p}\right)}^{n-2})(x_{0}).{\left({\left(M_{\alpha ,r}^{p}\right)}^{n-2}\right)}^{\prime }(x_{0}),=M_{\alpha ,r}^{p^{\prime }}({\left(M_{\alpha ,r}^{p}\right)}^{n-1}(x_{0})).M_{\alpha ,r}^{p^{\prime }}({\left(M_{\alpha ,r}^{p}\right)}^{n-2}(x_{0})).M_{\alpha ,r}^{p^{\prime }}({\left(M_{\alpha ,r}^{p}\right)}^{n-3}(x_{0}))\ldots M_{\alpha ,r}^{p^{\prime }}(M_{\alpha ,r}^{p}(x_{0})).(M_{\alpha ,r}^{p^{\prime }}(x_{0})).$$

Since ${\left(M_{\alpha ,r}^{p}\right)}^{k}(x_{0})=x_{k}$, ∀k∈N, that is, $(M_{\alpha ,r}^{p})(x_{0})=x_{1}$, ${\left(M_{\alpha ,r}^{p}\right)}^{2}(x_{0})=x_{2}=(M_{\alpha ,r}^{p})(x_{1})$, ${\left(M_{\alpha ,r}^{p}\right)}^{3}(x_{0})=x_{3}=(M_{\alpha ,r}^{p})(x_{2})$, and so on. Then, substituting in (23), we obtain

(24) $${\left({\left(M_{\alpha ,r}^{p}\right)}^{n}\right)}^{\prime }(x_{0})=M_{\alpha ,r}^{p^{\prime }}(x_{n-1}).M_{\alpha ,r}^{p^{\prime }}(x_{n-2}).M_{\alpha ,r}^{p^{\prime }}(x_{n-3})\ldots M_{\alpha ,r}^{p^{\prime }}(x_{1}).M_{\alpha ,r}^{p^{\prime }}(x_{0}),{\left({\left(M_{\alpha ,r}^{p}\right)}^{n}\right)}^{\prime }(x_{0})=\prod_{i=0}^{n-1}M_{\alpha ,r}^{p^{\prime }}(x_{i}).$$

Then, from (22) and (24), we obtain

(25) $$\mu =\lim_{n\to \infty }\frac{\kappa^{n}}{n}\ln|\prod_{i=0}^{n-1}M_{\alpha ,r}^{p^{\prime }}(x_{i})|=\lim_{n\to \infty }\frac{\kappa^{n}}{n}\sum_{i=0}^{n-1}\ln|M_{\alpha ,r}^{p^{\prime }}(x_{i})|,$$

where the parameter *p* stands for the orbit of period-*p*, *n* represents number of iterations and the Lyapunov exponent *μ* depends on $x_{0}$ and the kicked parameter *k* and the average Lyapunov exponent is given by (25) as the limit exists. If the orbit of the system approaches to periodic points of period-1,2,3,…, that is, a fixed point $x^{⁎}$ of period-1,2,3,…, then, from (25), we can say

(26) $$\mu =\kappa \ln|M_{\alpha ,r}^{\prime }(x^{⁎})|,\;\text{(for fixed points of period-1)}$$

(27) $$\mu =\kappa \ln|M_{\alpha ,r}^{2^{\prime }}(x^{⁎})|,\;\text{(for fixed points of period-2)}$$

(28) $$\mu =\kappa \ln|M_{\alpha ,r}^{3^{\prime }}(x^{⁎})|,\;\text{(for fixed points of period-3)}$$

(29) $$\ldots \;\ldots \;\ldots ,\mu =\kappa \ln|M_{\alpha ,r}^{p^{\prime }}(x^{⁎})|,\;\text{(for fixed points of period-}p\text{)}$$

and when the iterative orbit vibrates between the periodic points for *p*-times, then we obtain

(30) $$\mu =\frac{\kappa^{p}}{p}\sum_{i=0}^{p-1}\ln|M_{\alpha ,r}^{p^{\prime }}(x_{i})|.$$

Example 3.2 Let $\phi_{r}(x)=rx(1-x)$, be the classical nonlinear one-dimensional map and $M_{\alpha ,r}(x)$ be the superior dynamical system, where α∈(0,1) and r∈[0,4.22]. Then, using the hybrid chaos control procedure determine:

**(a)** the Lyapunov exponent for periodic state $x^{⁎}=0.6$ of period-1, when the kicked parameter κ=0.6176, α=0.9 and r=4.22,
**(b)** the Lyapunov exponent for periodic state $x^{⁎}=0.87$ of period-2, when the kicked parameter κ=0.8699, α=0.9 and r=4.22,

*Solution.***(a)** Let $M_{\alpha ,r}(x)=(1-\alpha )x+\alpha \phi_{r}(x)$ be the superior dynamical system, where $\phi_{r}(x)=rx(1-x)$ be the one-dimensional nonlinear map, then the control procedure with the kicked parameter *κ* is

(31) $$\kappa M_{\alpha ,r}(x)=\kappa [(1-\alpha )x+\alpha rx(1-x)],$$

where r∈[0,4.22] and α∈(0,1). Then, taking the derivative in (31), we get

(32) $$\kappa M_{\alpha ,r}^{\prime }(x)=\kappa (1-\alpha )+\kappa \alpha r(1-2x).$$

Now, to examine the stability behavior using Lyapunov exponent for $x^{⁎}=0.6$ of period-1 in the full range of the logistic parameter *r*, let us substitute α=0.9, κ=0.6176 and 0<r≤4.22 in (32):

(33) $$\kappa M_{\alpha ,r}^{\prime }(x)=0.6176(1-0.9)+0.6176\times 0.9\times 4.22(1-2\times 0.6),=0.6176\times 0.1-0.6176\times 0.9\times 4.22\times 0.2,=-0.4074.$$

Then, from equation (26), we obtain

(34) $$\mu =\ln|\kappa M_{\alpha ,r}^{\prime }(0.6)|=\ln|-0.4074|=-0.8979.$$

**Figure 19:**
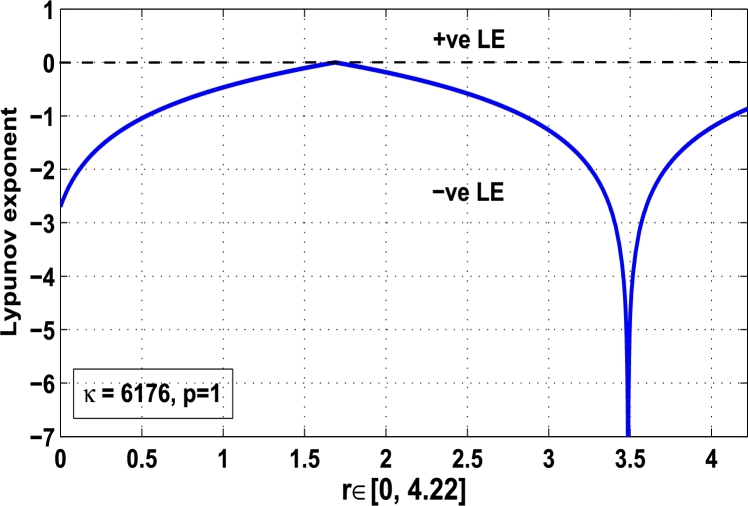
Kicked Lyapunov exponent plot of the system *M*_*α*,*r*_(*x*) for the fixed point *x*^⁎^ = 0.6 of period-1 when *κ* = 0.6176.

Thus, the Lyapunov exponent is negative for $x^{⁎}=0.6$ of period-1 with the kicked parameter value κ=0.6176, that means, the dynamics for 0<r≤4.22 is fully stable in the system as shown in Fig. 19.

**(b)** Similarly, taking the periodic point $x^{⁎}=0.87$ of period-2, κ=0.8699, α=0.9 and 0≤r≤4.22 and substituting in (19), we obtain

(35) $$\kappa M_{\alpha ,r}^{2^{\prime }}(0.87)=0.0607.$$

Then, using equation (35) and (27) for the fixed point of period-2, we get

(36) $$\mu =\ln|\kappa M_{\alpha ,r}^{2^{\prime }}(0.87)|=\ln|0.0607|=-2.8018.$$

**Figure 20:**
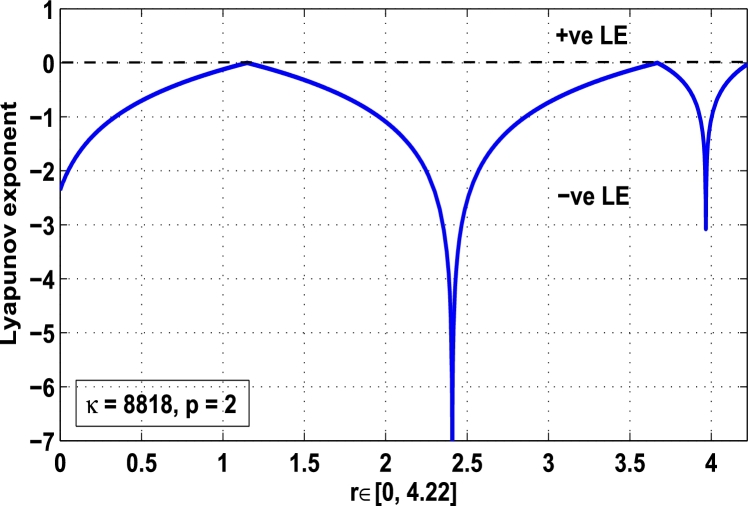
Kicked Lyapunov exponent plot of the system *M*_*α*,*r*_(*x*) for the fixed point *x*^⁎^ = 0.87 of period-2 when *κ* = 0.8818.

**Figure 21:**
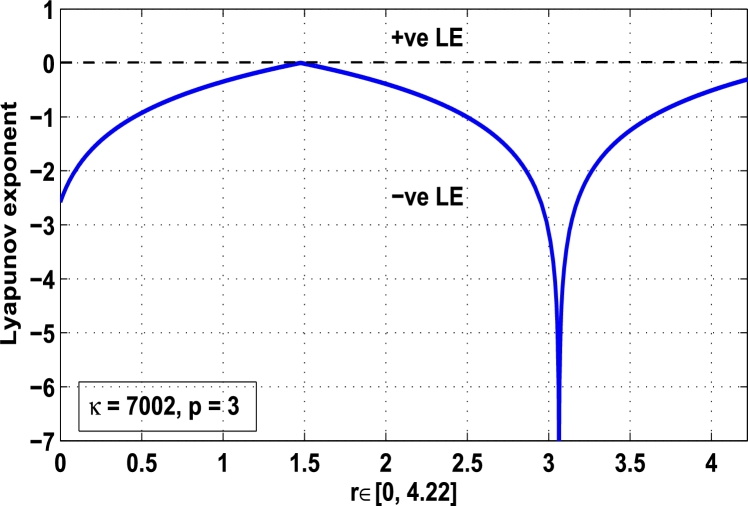
Kicked Lyapunov exponent plot of the system *M*_*α*,*r*_(*x*) for the fixed point *x*^⁎^ = 0.7 of period-3 when *κ* = 0.7002.

**Figure 22:**
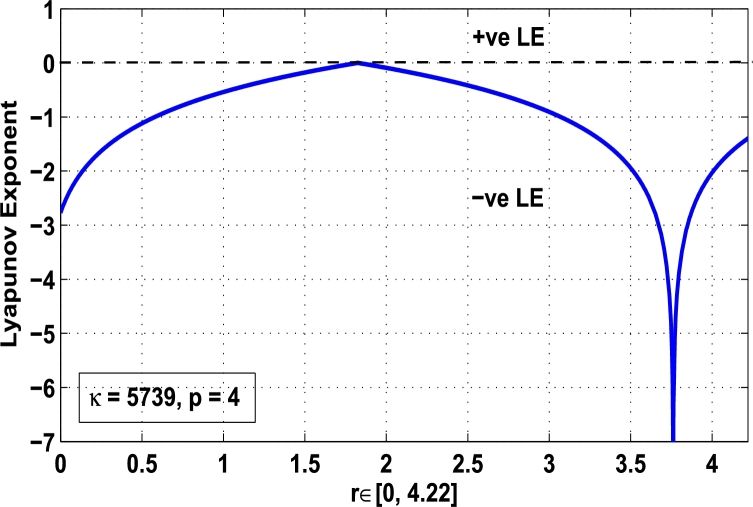
Kicked Lyapunov exponent plot of the system *M*_*α*,*r*_(*x*) for the fixed point *x*^⁎^ = 0.51 of period-4 when *κ* = 0.5739.

Thus, the Lyapunov exponent value for $x^{⁎}=0.87$ of period-2 is negative for the kicked parameter κ=0.8699 and hence the dynamics in the control procedure is stable at $x^{⁎}=0.87$ in r∈[0,4.22] as shown in Fig. 20. Figure 19, Figure 20, Figure 21, Figure 22 show the stability behavior using Lyapunov exponent values in the periodic orbits of period: one, two, three and four for the dynamical system $M_{\alpha ,r}(x)$ when they are kicked with the parameter κ=0.6176,0.8699,0.7002, and 0.5739, respectively. It is seen that in each case the Lyapunov trajectory converges to negative value in the full range of *r*. The maximum Lyapunov exponent for the fixed $x^{⁎}=0.6$ of period-1 approaches to μ=−0.8979 as shown in Fig. 19, for $x^{⁎}=0.87$ of period-2 approaches to −0.1018 as shown in Fig. 20, for $x^{⁎}=0.7$ of period-3 approaches to −0.3231 as shown in Fig. 21, and for $x^{⁎}=0.51$ of period-4 approaches to −1.4018 as shown in Fig. 22.

**Figure 23:**
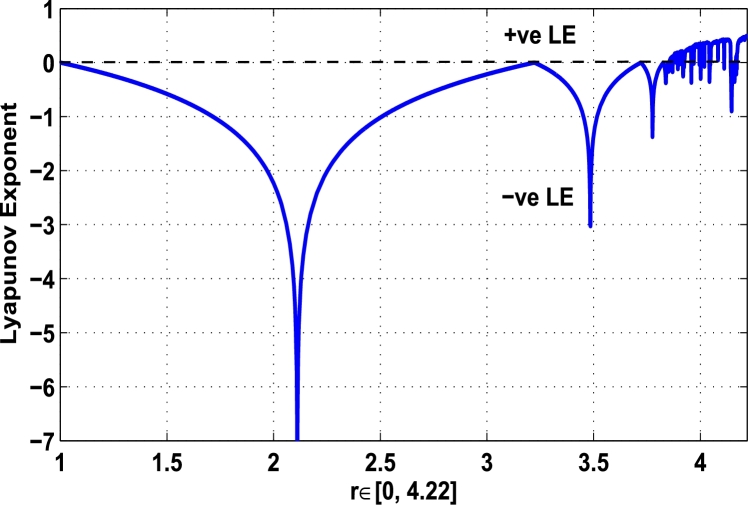
Standard Lyapunov exponent spectrum for *M*_*α*,*r*_(*x*) for 0 ≤ *r* ≤ 4.22 and *α* = 0.9.

**Figure 24:**
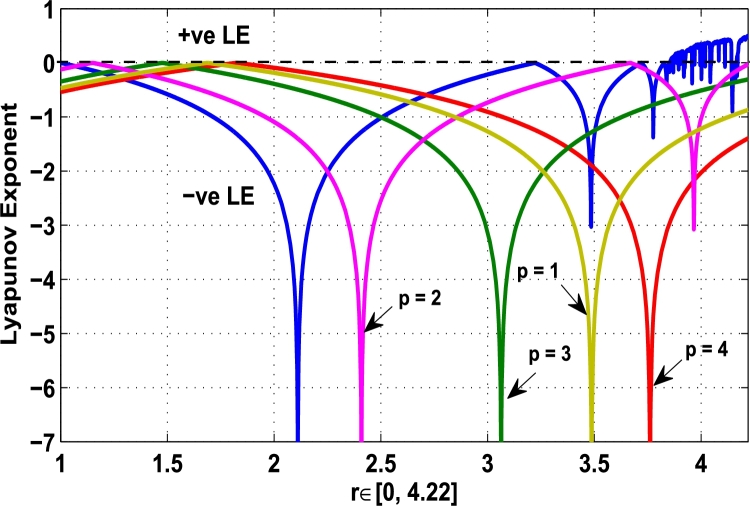
Standard Lyapunov exponent versus Kicked Lyapunov exponent trajectories for period-1, 2, 3, and 4.

In Fig. 23, the Lyapunov exponent trajectory is drawn for the superior dynamical system $M_{\alpha ,r}(x)$ in which the spectrum first approaches to negative values and then approaches to positive values as *r* approaches to 4.22. Finally, in Fig. 24 a comparative analysis versus original Lyapunov spectrum and the kicked Lyapunov spectrums for the orbits of period: one, two, three, and four are described.

### 3.3 Comparative analysis

Since the publication of the research of Ott et al. [1] in 1990, there has been a great study in the development of techniques for the control of various chaotic phenomena. Some of these methods are like, changing of the system parameter method, applying a damper, Pyragas method, stabilizing unstable periodic orbits, occasional proportional feedback, synchronization, etc. But our result depends on the kicked control parameter *κ*, parameter *α*, and *r* which make it unique from other control systems. Therefore, as compared to existing results it may benefit in various applications and provide superior results.

## 4 Conclusion

In this article, a novel hybrid chaos stability procedure with the composition of the superior feedback system and the kicked parameter perturbations has been developed. The numerical and experimental analysis show that this strategy may works more effectively to control chaos in dynamics. Throughout, the article it is described that the superior orbit ${\left\{x_{n}\right\}}_{n\in N}$ is kicked with the parameter κ∈(0,1) once at every $p^{th}$ iterations of the system. The outcomes are taken as:

1. In Section 2, a hybrid chaos control procedure is derived for discrete one-dimensional nonlinear equations which leads to strong stability in chaos through periodic orbits of order-*p*. Therefore, the system describes that the kicked control $x_{i}\to \kappa x_{i}$ after each $p^{th}$ iteration of the system $x_{n+1}=M_{\alpha ,r}(x_{n})$ control the system at periodic state of order-*p*.
2. The control curves for the system $F_{\alpha ,r}^{p}(x)=x.M_{\alpha ,r}^{p^{\prime }}(x_{n})/M_{\alpha ,r}^{p}(x_{n})$ versus x∈[0,1], are plotted for p=1,2,3,4,5 and 6. It is examined that when the value of the function $F_{\alpha ,r}^{p}(x)$ approaches between −1 and 1, then the hybrid chaos control system can stabilize the chaos at orbits of order *p*. Further, the time-series orbit diagrams are also generated for some particular values of *κ* which stabilizes to the periodic fixed points of order *p* as discussed in control curves.
3. In Section 3, Example 3.1 determines the control to chaos using the hybrid chaos control procedure where the unstable orbit settles down in the stable states of orders 1 and 2 for the kicked parameter κ=0.6176 and 0.8818, respectively. Further, the stability is shown using the period-doubling bifurcation diagram in which the aperiodicity completely approaches to the stable periodic states. Example 3.2 examines the stability in chaos through the Lyapunov exponent value for the fixed orbits of order 1 and 2 where the system is kicked with the parameter *κ*.
4. Figure 1, Figure 2, Figure 3, Figure 4, Figure 5, Figure 6, determine the regime of arbitrary points of order *p* in the closed interval [0, 1] which satisfies the stability condition (7), and for the values of $x^{⁎}$ and *κ* the original dynamical system (10) gets stability in fixed points of periodic order 1 to 6 as shown in Figure 7, Figure 8, Figure 9, Figure 10, Figure 11, Figure 12, Figure 13, Figure 14. Figure 15, Figure 16, Figure 17, Figure 18 shows the bifurcation representation in the unstable orbit settle down in a stable state depending on the kicked parameter *κ*.
5. The Lyapunov exponent another generic property in nonlinear dynamical systems, often used whether or not the system is chaotic depending on the initial parameter is determined. The findings on the Lyapunov exponent are examined for the hybrid chaos control procedure followed by the particular example in which the Lyapunov exponent trajectory approaches to negative value. Figure 19, Figure 20, Figure 21, Figure 22, Figure 23, Figure 24 show the kicked Lyapunov exponent for $M_{\alpha ,r}(x)$ in which the trajectory always approaches to a negative value.
6. Finally, considerable research has been done to control chaos in recent decades. Various practical methods have been applied in cardiology, population models, communications, physics laboratories, biochemistry, transportation problems, and turbulence. Therefore, this method may fit into the various applications of real life.

## Funding statement

Prof. Mohammad Sajid is supported by the Deanship of Scientific Research, Qassim University, Saudi Arabia for funding of APC for this publication.

## CRediT authorship contribution statement

**Ashish:** Writing – original draft, Methodology, Investigation, Formal analysis, Conceptualization. **Mohammad Sajid:** Writing – review & editing, Methodology, Funding acquisition, Conceptualization.

## Declaration of Competing Interest

The authors declare that they have no known competing financial interests or personal relationships that could have appeared to influence the work reported in this paper.

## Data Availability

Data included in article/supplementary material/referenced in article.
